# Early Engagement of Parietal Cortex for Subtraction Solving Predicts Longitudinal Gains in Behavioral Fluency in Children

**DOI:** 10.3389/fnhum.2020.00163

**Published:** 2020-05-26

**Authors:** Macarena Suárez-Pellicioni, Ilaria Berteletti, James R. Booth

**Affiliations:** ^1^Department of Educational Studies in Psychology, Research Methodology, and Counseling, The University of Alabama, Tuscaloosa, AL, United States; ^2^Educational Neuroscience Program, Gallaudet University, Washington, DC, United States; ^3^Department of Psychology and Human Development, Vanderbilt University, Nashville, TN, United States

**Keywords:** arithmetic, subtraction, fluency, fMRI, longitudinal, children

## Abstract

There is debate in the literature regarding how single-digit arithmetic fluency is achieved over development. While the Fact-retrieval hypothesis suggests that with practice, children shift from quantity-based procedures to verbally retrieving arithmetic problems from long-term memory, the Schema-based hypothesis claims that problems are solved through quantity-based procedures and that practice leads to these procedures becoming more automatic. To test these hypotheses, a sample of 46 typically developing children underwent functional magnetic resonance imaging (fMRI) when they were 11 years old (time 1), and 2 years later (time 2). We independently defined regions of interest (ROIs) involved in verbal and quantity processing using rhyming and numerosity judgment localizer tasks, respectively. The verbal ROIs consisted of left middle/superior temporal gyri (MTG/STG) and left inferior frontal gyrus (IFG), whereas the quantity ROIs consisted of bilateral inferior/superior parietal lobules (IPL/SPL) and bilateral middle frontal gyri (MFG)/right IFG. Participants also solved a single-digit subtraction task in the scanner. We defined the extent to which children relied on verbal vs. quantity mechanisms by selecting the 100 voxels showing maximal activation at time 1 from each ROI, separately for small and large subtractions. We studied the brain mechanisms at time 1 that predicted gains in subtraction fluency and how these mechanisms changed over time with improvement. When looking at brain activation at time 1, we found that improvers showed a larger neural problem size effect in bilateral parietal cortex, whereas no effects were found in verbal regions. Results also revealed that children who showed improvement in behavioral fluency for large subtraction problems showed decreased activation over time for large subtractions in both parietal and frontal regions implicated in quantity, whereas non-improvers maintained similar levels of activation. All children, regardless of improvement, showed decreased activation over time for large subtraction problems in verbal regions. The greater parietal problem size effect at time 1 and the reduction in activation over time for the improvers in parietal and frontal regions implicated in quantity processing is consistent with the Schema-based hypothesis arguing for more automatic procedures with increasing skill. The lack of a problem size effect at time 1 and the overall decrease in verbal regions, regardless of improvement, is inconsistent with the Fact-retrieval hypothesis.

## Introduction

Failing math in sixth grade is a significant predictor of not graduating from high school (Belfanz et al., 2007) and math ability at age 7 predicts socioeconomic status at age 42 (Ritchie and Bates, 2013). Gaining fluency in solving single-digit arithmetic facts is an important milestone in mathematical development, freeing up working memory (Geary, 1994) and scaffolding higher-level math skills (Price et al., 2013). Despite the importance of math fluency, the neurocognitive mechanisms predicting its successful development are poorly understood.

Two hypotheses have been formulated to explain fluency development of subtraction problems. According to the Fact-retrieval hypothesis, children initially rely on slow procedures, such as counting, to solve single-digit subtractions, but with the repeated use of procedures, the problem (i.e., 5−2) and its solution (i.e., 3) are stored in long-term memory, so children shift toward retrieval (Ashcraft, 1982; Siegler, 1987). Some behavioral studies interpret the response times patterns shown by children as young as 5 years old as evidence in favor of the retrieval strategy for solving the majority of subtraction problems (Siegler, 1987). Others, relying on self-report, found that 5th graders use more retrieval and less counting to solve subtraction problems as compared to 3rd graders, who reported using more procedures (Caviola et al., 2018). According to this hypothesis, educated adults have had enough experience with arithmetic to be able to retrieve single-digit subtractions directly from memory (Siegler, 1989; Geary et al., 1993).

On the other hand, Baroody (1983) claimed that Ashcraft (1982)’s classification of retrieval as being fast and procedures as being slow was a biased assumption, and that faster response times (RTs) over development could also be explained by procedures becoming more automatic, which is the core assumption of the Schema-based hypothesis. According to Baroody (1983), children move from initial reliance on less efficient procedures such as counting to more efficient procedures including principles, heuristics or rules (e.g., N + 0 = N; N × 0 = 0; N – N = 0; N−1 or N + 1 = number before or after N, respectively, in the counting sequence). Studies have suggested that procedures are solved more efficiently throughout elementary school (Woods et al., 1975) but the application of procedures seems to depend on problem type. Barrouillet et al. (2008) showed that 3rd graders reported using retrieval less frequently to solve subtractions (i.e., 19%) as compared to additions (65%; Barrouillet and Lépine, 2005) and that the retrieval of subtractions was limited to problems having a remainder of 1. Studies with adults have shown that university students retrieved only 71% (Geary et al., 1993) and 57% (Campbell and Xue, 2001) of subtractions. Procedures that adults rely on include addition reference (i.e., referring to 4 + 5 = 9 to solve 9−4 = 5; Peters et al., 2012; Chang et al., 2015), counting down (i.e., 9−2 = eight, seven) and reconstruction (i.e., for 9−4, do 10−4 = 6; 6−1 = 5) (Kirk and Ashcraft, 2001; Seyler et al., 2003; LeFevre et al., 2006). Studies have shown that the efficiency with which complex subtractions are solved improve even in adulthood, with older adults (i.e., 61−80 years old) being faster in applying borrowing as compared to younger adults (i.e., 18−38 years old) (Geary et al., 1993). Núñez-Peña et al. (2015) compared low and high skilled participants in a subtraction verification task in which participants reported the strategy they used to solve the problem. They found that while the two groups did not differ in the frequency of procedures vs. retrieval use, the high skilled individuals were faster and less error-prone than the less skilled ones when solving the trials for which they had reported procedural use, suggesting greater efficiency in carrying out those procedures.

Our knowledge of how subtraction problems are solved comes from behavioral studies using RTs and self-reported measures (Siegler, 1989). However, evidence has suggested that inferring mental processes from RTs can provide misleading information (Siegler, 1989) and that introspection of performance might be limited when a participant is asked to describe the strategy used when the process is fast and automatic (Ericsson and Simon, 1993; Lefevre et al., 1996). As suggested by Fayol and Thevenot (2012), participants may report using retrieval because procedures were implemented so automatically that they were not even aware of having used them. Others have claimed that the simple fact of asking about the strategies being used may alter the cognitive process, biasing participants to use those strategies that they think might be expected by the examiner (Kirk and Ashcraft, 2001). Functional magnetic resonance imaging (fMRI) can help to overcome the limitations of response times and self-reported measures by providing evidence of the underlying neurocognitive mechanisms associated with the development of subtraction fluency. Finding verbal regions of the brain to be associated with subtraction fluency gains would be compatible with the Fact-retrieval hypothesis, whereas finding quantity regions to be associated with subtraction fluency gains would be supportive of the Schema-based hypothesis. Rivera et al. (2005) found age-related increases in temporo-parietal regions, including left middle temporal gyrus (MTG) and supramarginal gyrus extending to the left intraparietal sulcus (IPS) and decreases in frontal regions such as inferior/middle frontal gyri (IFG/MFG), when 8- to 19-year-old participants solved a single-digit addition and subtraction task. Price et al. (2013) found that high school students with higher scores on a math test relied on brain regions associated with retrieval to solve single-digit additions and subtractions, whereas students with lower scores relied on brain regions associated with procedures in right IPS. Looking at the problem size effect in the brain, De Smedt et al. (2011) found that 10−12-year-old children with typical fluency relied less on quantity mechanisms in right IPS to solve small additions and subtractions, whereas children with low fluency relied on this region to solve all problems regardless of size. Polspoel et al. (2017) found that single-digit multiplications and subtractions that were reported to be solved by retrieval by 4th graders activated temporal cortex regions associated with retrieval. However, these studies have investigated brain activation by averaging across different operation types (i.e., addition and subtraction, usually). Neuroimaging evidence has shown that different operations recruit distinct neural networks (Arsalidou and Taylor, 2011; Rosenberg-Lee et al., 2011), so examining brain activation across different operations may have washed away subtraction-specific effects in the brain.

Other fMRI studies have compared subtraction processing with addition or multiplication. They have found that while additions (Rosenberg-Lee et al., 2015; Evans et al., 2016) and multiplications (Prado et al., 2011) activated verbal regions associated with retrieval, solving subtractions activated the parietal cortex, associated with procedures. Rosenberg-Lee et al. (2011) compared brain activations between single-digit addition vs. subtractions and found greater IPS activation for the latter. Prado et al. (2014) reported that children showed greater activation in the right parietal cortex when solving single-digit subtractions compared to multiplications, and this difference increased with more years of math instruction. While Prado’s study can be interpreted as evidence supporting the Schema-based hypothesis, they studied maturation-related effects in the brain in a cross-sectional design that showed only a modest behavioral improvement. Concerns have been raised with the use of cross-sectional data to answer developmental questions, due to the large variability introduced by studying children from different ages, which might fail to detect or falsely suggest changes over time (Casey et al., 2005). Longitudinal studies overcome these limitations by studying the same cohort of individuals at two different time points, and constitute the recommended design (Karmiloff-Smith, 2010).

Using a longitudinal design, Artemenko et al. (2018) found that children showed reductions in frontal cortex, including MFG, from 6th to 7th grade when solving two-digit subtractions, which was accompanied by an improvement in accuracy. The reduction in frontal cortex was interpreted as less reliance on cognitive control. However, this result does not clarify whether it is fact retrieval or the use of procedures that become more efficient over time. Similar inconclusive results were found in studies showing age-related increases in both bilateral IPS and left MTG, areas associated with the use of procedures and retrieval, respectively (Rivera et al., 2005; Chang et al., 2016).

To the best of our knowledge, neuroimaging studies have not yet provided a clear picture of the underlying mechanisms responsible for fluency development in subtraction. The objective of this study was to fill this gap in the literature by answering the questions: Can reliance on verbal vs. quantity mechanisms at time 1 predict longitudinal gains in subtraction fluency, and how do these mechanisms change over time with improvement in subtraction fluency? In order to have stronger evidence for the involvement of verbal vs. quantity mechanisms, regions of interest (ROIs) were independently localized for each participant using rhyming and numerosity judgment localizer tasks, respectively. We identified ROIs implicated in the storage of phonological representations in the left MTG/STG (e.g., Prado et al., 2011), and in the access to those representations in the left IFG (e.g., Prado et al., 2011). We also localized ROIs in bilateral IPL/SPL implicated in quantity representations (e.g., Dehaene et al., 2003), and in the access of those representations in the bilateral MFG/right IFG (e.g., Arsalidou and Taylor, 2011). We then defined the extent to which children relied on verbal vs. quantity mechanisms to solve subtractions by selecting the 100 voxels showing maximal activation from each ROI, separately for small and large subtractions.

We aimed to study whether brain activation at time 1 predicts subtraction fluency gains as well as whether these neurocognitive mechanisms changed over time with fluency gains. Finding that brain activation in bilateral parietal cortex predicts the fluency gains would be compatible with both hypotheses, given that children may continue to rely on procedures that become more automatic with experience (i.e., Schema-based hypothesis), or may later shift toward retrieval (i.e., Fact-retrieval hypothesis). According to the Schema-based hypothesis, we expected to see increases in parietal cortex activation over time, suggesting that children continue to rely on procedures. However, we also expected to see decreases in bilateral MFG/right IFG over time, suggesting that procedures become more automatic (see arrow A in Figure 5; Schema-based). According to the Fact-retrieval hypothesis, we expected to see decreases in parietal cortex and increases in temporal cortex over time. It is possible that this process is accompanied by increases in left IFG activation over time, given that the implementation of retrieval strategy might be effortful in its early stages (Geary et al., 1996a; i.e., see arrow B in Figure 5; Fact-retrieval). Finally, there is a third possibility. Considering evidence suggesting that by age 10 retrieval may be the dominant strategy to solve single-digit arithmetic problems (Ashcraft and Fierman, 1982), it might be the case that children have already shifted toward retrieval at time 1, in which case we expect to see activation in temporal cortex early on to predict fluency gains. In this scenario, we expect children to show increases in temporal cortex activation over time, suggesting that they build their storage of subtraction facts in long-term memory. This might be accompanied by decreases in left IFG over time, suggesting that the retrieval becomes less effortful as the representations become more robust (Prado et al., 2014; see arrow C in Figure 5; Fact retrieval).

## Materials and Methods

### Participants

#### Whole Sample

Sixty-five 3rd to 8th graders were recruited from schools in the Chicago metropolitan area to participate in the study. This dataset has been deposited in OpenNeuro (10.18112/openneuro.ds001486.v1.1.0) and a detailed description of the dataset is provided in Suárez-Pellicioni et al. (2019b). Timepoint 1 of this dataset is the basis of other publications by our research group, including (Berteletti et al., 2014; Demir-Lira et al., 2014, 2015; Prado et al., 2014; Berteletti and Booth, 2015a, b; Demir-Lira et al., 2016). The longitudinal data of this dataset is the basis of other publications including Suárez-Pellicioni and Booth (2018), Suárez-Pellicioni et al. (2018), Suárez-Pellicioni et al. (2019a). None of them have looked at longitudinal gains in subtraction fluency, which constitutes the objective of this study.

All participants were native English speakers, right-handed, were free of past and present psychiatric disorders including Attention Deficit Hyperactivity Disorder (ADHD), neurological disease or epilepsy. According to parental report, no participant had hearing impairments, uncorrected visual impairment, was born prematurely (less than 36 weeks), was taking medication affecting the central nervous system or had any contraindication for being scanned, such as having braces. Participants had no history of intellectual deficits, all of them scoring above 85 standard score (hereinafter, SS) on the Full IQ scale of the Wechsler Abbreviated Scale of Intelligence – WASI (Weschler, 1999). All participants scored above 71 SS on the Math Fluency subtest from the Woodcock-Johnson III Test of Achievement (WJ-III; Woodcock et al., 2001) and above 85 SS on the average of Word Attack and Word Identification tests of the WJ-III. Children and their parents or guardians provided written consent to participate in the study. Parents were compensated $20 per hour for their time. All experimental procedures were approved by the Institutional Review Board at Northwestern University.

Data from six participants had to be excluded because of having excessive movement in the scanner. Excessive movement was defined as more than 10% of the total volumes replaced or more than five consecutive volumes replaced in a given run (for more details, see Section “fMRI Data Analysis”). There was no correlation between number of volumes replaced and age (*r* = −0.12, *p* = 0.43) or improvement (*r* = 0.09, *p* = 0.51).

Data from another six participants were excluded for showing accuracy below 50% in the small condition of the subtraction task solved inside the scanner either at time 1 or time 2 (for more specific information see section “Subtraction Task Behavioral Results”). Six additional participants had to be excluded for showing accuracy below 33% for the control condition (i.e., blue square). One participant was excluded for being left-handed.

The final sample consisted of 46 participants^1^ who were tested longitudinally, with sessions being approximately 2 years apart. More detailed information about the sample is given in Table 1.

**TABLE 1:** Demographic characteristics and standardized scores.

	Whole sample (*n* = 46)	Improvers (*n* = 23)	Non-improvers (*n* = 23)	Group differences
Age at T1 session (years)	11.2 (1.5)	11.1 (1.6)	11.3 (1.5)	*t*(44) = −0.56, *p* = 0.58
Age at T2 session (years)	13.4 (1.6)	13.3 (1.8)	13.6 (1.6)	*t*(44) = −0.60, *p* = 0.55
Time between sessions (years)	2.1 (0.2)	2.1 (0.2)	2.2 (0.2)	*t*(44) = −0.63, *p* = 0.54
Female/male ratio	25/21	15/8	10/13	*X*^2^ = 2.20, *p* = 0.14
Reading at T1 (SS)	107.0 (10.3)	107.7 (10.6)	106.3 (10.3)	*t*(44) = 0.45, *p* = 0.65
Verbal WM at T1 (SS)	103.0 (13.4)	103.8 (15.6)	102.3 (11.1)	*t*(44) = 0.39, *p* = 0.70
Visuo-spatial WM at T1 (SS)	106.0 (13.0)	106.9 (15.0)	104.9 (10.8)	*t*(44) = 0.52, *p* = 0.60
Verbal IQ at T1 (SS)	114.0 (16.0)	115.7 (16.3)	111.3 (15.3)	*t*(44) = 0.94, *p* = 0.35
Performance IQ at T1 (SS)	110.2 (15.2)	113.0 (16.1)	107.5 (14.1)	*t*(44) = 1.23, *p* = 0.22

*Participant’s age at each time point, time between sessions, number of females and standard scores (i.e., adjusted for age norms) in reading skill, working memory (WM) and intelligence (IQ) at time 1 for the whole sample (*n* = 46), for the improvers (*n* = 23), and non-improvers (*n* = 23). The last column indicates statistical values for the comparison between improvers and non-improvers. SS, Standard score; T1, Time 1; T2, Time 2. See section “Standardized Measures” for a description of the tests used to measures reading skill, verbal WM, visuo-spatial WM, verbal IQ, and performance IQ.*

#### Improvement Groups

Two groups were created based on improvement on the subtraction task solved inside the scanner: improvers and non-improvers (see section “Experimental Task: Single Digit Subtraction” for a description of the subtraction task and its conditions). To form the groups, we first calculated the difference in means of response times between time points (i.e., Time 2-Time 1) for large subtractions. In order to account for initial differences in performance, we regressed time 1’s response times out from the difference score, saving the residuals. These residuals represented the difference in response times after initial levels have been accounted for. Then, we created two groups based on the median-split of these residuals: improvers (*n* = 23) and non-improvers (*n* = 23). The decision of using large subtractions was made given the simplicity of the small subtractions in our study, with half of the problems having a remainder of 1 (e.g., 3 – 2 = 1), the largest remainder being 3 (e.g., 5 – 2 = 3), and that 40% of the problems included minuends smaller or equal 5. More detailed information about these two groups is shown in Table 1. The two groups did not differ in age at time 1, age at time 2, time between sessions, sex distribution, reading skill, verbal WM, visuo-spatial WM, verbal IQ, or performance IQ (all *p-*Values above.22; all measured using age-adjusted norms). For more information on differences in performance between these groups see section “Improvement Groups’ Performance” and Figure 6.

### Standardized Measures

Reading skill was measured as the average of standard scores on the Word Attack and the Word Identification subtest from the Woodcock-Johnson III Test of Achievement (WJ-III; Woodcock et al., 2001) at time 1. The Word Attack requires oral reading of pseudo-words, while the Word Identification test requires oral reading of isolated letters and real words.

Verbal working memory (WM) was measured by the Listening Recall subtests of the Automated Working Memory Assessment (AWMA; Alloway et al., 2007). This subtest requires children to decide whether a sentence is true or false and also to remember the final word of the sentence. Thus, children are asked to store the final word of the sentence, as they process an increasing number of new sentences. The item is scored as correct if children recall the correct word or words in the correct order.

Visuo-spatial WM was measured with the Spatial Recall subtest of the AWMA (Alloway et al., 2007). In this test, children view pictures of two shapes where the shape on the right has a red dot near it and they need to identify whether the shape on the right is the same as the shape on the left when rotated in two dimensions, or whether it is the mirror image. At the end of the trial, individuals are asked to remember the position of the red dot and to answer by pointing to a picture with three possible positions marked. The number of shape pairs to be compared increases as children proceed through the test, and participants must recall the correct position of all the red dots in the correct temporal order.

Intelligence was measured using both the Verbal and Performance subscales of the Wechsler Abbreviated Scale of Intelligence (WASI; Weschler, 1999). Verbal IQ was measured with the Vocabulary subtest, in which the participants have to define words, and with the Similarities subtest, in which the participants are presented with two words that represent common objects or concepts and they have to describe how they are similar. Performance IQ was measured with Block Design and Matrix Reasoning subtests of the WASI. The Block Design requires the participants to use red-and-white blocks to re-create, within a specified time limit, a model design. In the Matrix Reasoning subtest, participants view an incomplete series or matrix and select the response option that completes it logically.

### Scanner Tasks

#### Rhyming Judgment Localizer Task to Identify Verbal Regions in the Brain

**FIGURE 1:**
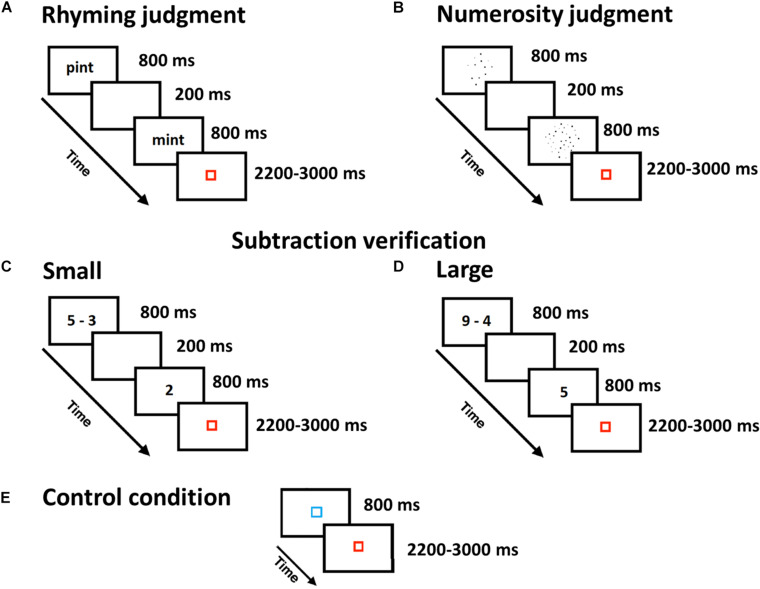
Experimental tasks and their timing. Localizer task: **(A)** The rhyming judgment task was used to identify verbal regions of the brain in which participants had to respond whether pairs of words rhymed or not. **(B)** The numerosity judgment task was used to identify quantity regions of the brain in which participants had to indicate which of the two sets of dots had a greater number. Subtraction task: Single-digit verification task, including **(C)** small and **(D)** large subtractions. **(E)** Control condition common to all tasks, in which participants had to press a button when the blue square turned red.

In the rhyming judgment task, two written monosyllabic English words were sequentially presented and participants had to decide whether the words rhymed or not. To ensure that participants relied on phonology to solve the task, and not orthography, we created four conditions in which pairs of words had: (1) similar orthography and similar phonology (i.e., O + P +; e.g., *dime–lime*; 12 trials); (2) similar orthography but different phonology (i.e., O + P-; e.g., *pint–mint;* 10 trials); (3) different orthography but similar phonology (i.e., O−P +; e.g., *jazz–has;* 10 trials); (4) different orthography and different phonology (i.e., O−P−; e.g., *press–list;* 14 trials). The O + P + and O−P− constituted the non-conflicting conditions, given that orthographic information was consistent with the right answer, whereas the O-P + and O + P− conditions constituted the conflicting conditions because orthographic information was inconsistent with the right answer. Figure 1A shows an example of an O + P- condition of the rhyming judgment task. The control condition consisted of a blue square that was presented for the same duration as the experimental conditions and children were asked to press a button when the square turned red (Figure 1E). Stimuli were presented in a single run, lasting approximately 7 min. All participants received trials in the same order.

#### Numerosity Judgment Localizer Task to Identify Quantity Regions in the Brain

Participants were sequentially presented with two dot arrays and their task was to decide which of them had more dots. The task comprised 24 easy (i.e., compare 12 vs. 36 dots), 24 medium (i.e., 18 vs. 36), and 24 hard (i.e., 24 vs. 36 dots) trials. The first dot array was composed of the larger number of dots in half of the trials, while it was composed of the smaller number of dots in the other half. To ensure that participants’ judgments were based on differences in quantity rather than cumulative surface area, the distribution of dot sizes was biased toward smaller dots in large arrays and bigger dots in small arrays. However, totally equating the cumulative surface area between small and large arrays by entirely biasing the distribution of single dot sizes (100% bias) may have led participants to use single dot sizes as a cue for their judgments. Therefore, we found a trade-off (50% bias) between equating as much as possible the cumulative surface areas and the distributions of single dot sizes in each pair. Figure 1B shows an example of an easy condition of the numerosity judgment task. The control condition consisted of a blue square that was presented for the same duration as the experimental conditions and children were asked to press a button when the square turned red (Figure 1E). Stimuli were divided into two runs, lasting approximately 4 min each. All participants received trials in the same order within each run.

#### Experimental Task: Single-Digit Subtraction

Participants were presented with a single-digit subtraction problem followed by a proposed solution and were asked to decide whether the proposed solution was true or false. Problems were broken down into small (Figure 1C) and large (Figure 1D) single-digit problems. Small subtractions (12 problems) were characterized by having a small difference (i.e., 1, 2, or 3) between the first and second term of the subtraction (e.g., 3 - 2), regardless of the first term size. In large subtractions (12 problems), the first term was relatively large (i.e., 6, 7, 8, or 9), as was the difference between the first and second terms (i.e., 3, 4, 5, or 6; e.g., 9 - 4). Each problem was repeated twice with a true solution and once with a false solution, yielding a total of 72 trials. False solutions were constructed by adding 1 or 2 to the correct solution (e.g., 8 – 2 = 7), or by subtracting 1 from the correct solution (e.g., 8 – 5 = 2). Problems involving 0 (e.g., 3 – 3; 3 – 0) or 1 as the second operand (e.g., 3 - 1) and ties (e.g., 6 - 3) were only used in the practice session. The control condition consisted of a blue square that was presented for the same duration as the experimental conditions and children were asked to press a button when the square turned red (Figure 1E). Stimuli were divided into two runs, lasting approximately 4 min each. All participants received trials in the same order within each run.

### Experimental Protocol

First, informed consent was obtained from the children and their parents or guardians, and then standardized tests were administered. Children then had a practice session in which they practiced all trial types and learned to minimize head movement in a mock fMRI scanner. For the rhyming and numerosity localizer tasks, the practice session consisted of twelve trials of each condition. For the subtraction task, twenty-four problems with a correct proposed solution and 24 problems with a false proposed solution were included in the practice session. For all the tasks, the items used for the practice session were different from the ones used for the scanning session.

The actual scanning session took place within a week of the practice session. In the fMRI scanner, participants performed one run of the rhyming judgment task, two runs of the numerosity judgment task and two runs of the subtraction verification task. The order of the tasks and the runs was counterbalanced across participants. The timing and order of trial presentation were optimized for estimation efficiency using optseq2^2^. Behavioral responses were recorded using an MR-compatible keypad and participants responded with their right hand. Participants responded with their index finger if the two words rhymed, if the first array of dots had more dots, if the proposed solution for the subtraction problem was correct, or when the blue square from the control condition turned red. Participants used their middle finger if the two words did not rhyme, if the second array of dots had more dots, or if the proposed solution for the subtraction problem was incorrect. Stimuli were generated using E-prime software (Psychology Software Tools, Pittsburgh, PA, United States) and projected onto a screen that was viewed by the participants through a mirror attached to the head-coil.

### Stimulus Timing

Stimulus timing was identical for all tasks. A trial started with the presentation of a first stimulus (i.e., first word, first array of dots, or subtraction operation) for 800 ms followed by a blank screen for 200 ms. A second stimulus (i.e., second word, second array of dots, or proposed solution for the subtraction operation) was presented for 800 ms, and followed by a red fixation square for 200 ms. Variable periods of fixation, ranging from 2200 to 3000, were added after each trial in order to help with convolution, during which a red square was presented. Participants could respond as soon as the second word was presented until the beginning of the next trial. As for the control condition, the blue square was presented for 800 ms followed by a red fixation square lasting 2200-3000 ms. The run ended with 22 s of passive visual fixation in order to aid in deconvolution of the final trials.

### fMRI Data Acquisition

Images were collected using a Siemens 3T TIM Trio MRI scanner (Siemens Healthcare, Erlangen, Germany) at Northwestern University’s Center for Advanced MRI. The fMRI blood oxygenation level dependent (BOLD) signal was measured with a susceptibility weighted single-shot echo planar imaging (EPI) sequence. The following parameters were used: TE = 20 ms, flip angle = 80°, voxel size: 1.7 × 1.7 × 3 mm, matrix size = 128 × 120 × 37, field of view = 220 × 206.25 × 111 mm, slice thickness = 3 mm (0.48 mm gap), number of slices = 32, TR = 2000 ms. Before functional image acquisition, a high resolution T1 weighted 3D structural image was acquired for each subject, with the following parameters: TR = 2300 ms, TE = 3.36 ms, matrix size = 256 × 256, field of view = 240 mm, slice thickness = 1 mm, number of slices = 160.

### fMRI Data Analysis

#### Preprocessing

Data analysis was performed using SPM8^3^. The first six images of the run were discarded to allow for T1 equilibration effects. The remaining functional images were corrected for slice acquisition delays, realigned to the first image of the run to correct for head movements, and spatially smoothed with a Gaussian filter equal to twice the voxel size (4 × 4 × 8 mm^3^ full width at half maximum). Prior to normalizing images, we used ArtRepair (^4^ Mazaika et al., 2009) to identify outlier volumes with more than 1.5 mm in volume-to-volume movement in any direction, or with more than 4% deviation from the mean global signal. The outlier volumes were repaired by interpolation between the nearest non-outlier volumes. All participants had less than 10% of the total number of volumes replaced and less than 5 volumes replaced in a row. Interpolated volumes were then partially de-weighted when first-level models were calculated on the repaired images (Mazaika et al., 2007). Functional volumes were co-registered with the segmented anatomical image and normalized to the standard T1 Montreal Neurological Institute (MNI) template volume (normalized voxel size, 2 × 2 × 4 mm^3^).

#### fMRI Processing

Event-related statistical analysis was performed according to the general linear model. Activation was modeled as epochs with onsets time-locked to the presentation of the first stimulus in each trial. All epochs were convolved with a canonical hemodynamic response function. The time series data were high-pass filtered (1/128 Hz), and serial correlations were corrected using an autoregressive AR model. Considering that improvement groups did not significantly differ in accuracy at either time point (see section “Improvement Groups’ Performance” for more details) and in order to equate for power in the analysis, all children’s responses (i.e., correct and incorrect) were included in the model.

#### Regions of Interest Definition

Regions of interests were defined base on a sample of 40 participants. Six participants^5^ had to be excluded for ROI definition because of having low accuracy in the rhyming judgment task (*n* = 1) and due to excessive movement in both localizer tasks (*n* = 5). Five combined ROIs were created, combining functional and anatomical ROIs. These combined ROIs were created by identifying the regions showing activation for the rhyming and numerosity judgment localizer tasks within fronto-temporal and fronto-parietal anatomical regions, respectively. The rationale for using combined ROIs, instead of only anatomical ones, was to be more confident of the underlying cognitive mechanisms (i.e., verbal vs. quantity) engaged during subtraction solving.

**FIGURE 2:**
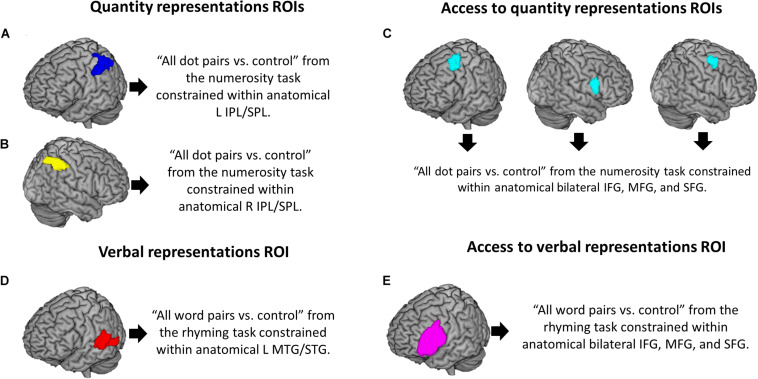
Visualization of regions of interest. Regions of the brain implicated in: quantity representations in **(A)** left and **(B)** right IPL/SPL; access to quantity representations in **(C)** bilateral MFG/right IFG (including left MFG, right IFG, and right MFG); **(D)** storage of verbal representations in left MTG/STG; and **(E)** access to verbal representations in left IFG.

To localize quantity regions in the brain we identified, for each participant, the voxels that showed greater activation for all dot pairs of the numerosity judgment task as compared to the control condition, at time 1. In a second-level analysis, these individual contrasts were submitted to a one-sample *t*-test across all participants. Given extensive evidence suggesting that the bilateral intraparietal sulci (IPS) is the crucial neural substrate for numerical magnitude processing (Pinel et al., 2001; Dehaene et al., 2003; Sokolowski et al., 2017), we used the bilateral IPL/SPL anatomical regions to ensure coverage of the IPS. We then constrained the brain activation elicited by the numerosity judgment localizer task within the anatomical bilateral IPL/SPL and took this combined ROI as the region responsible for quantity representations in left (Figure 2A) and right (Figure 2B) parietal cortices. All anatomical regions were defined using the anatomical automatic labeling (aal) template, which is part of the WFU pickatlas tool (Maldjian et al., 2003). Given previous evidence suggesting that the left IPL/SPL plays a crucial role in calculation (Simon et al., 2002; Rivera et al., 2005; Price et al., 2016), we considered left and right IPL/SPL as separate ROIs, in order to explore hemispheric differences.

To localize verbal regions in the brain we identified, for each participant, the voxels that showed greater activation for all word pairs of the rhyming judgment task as compared to the control condition, at time 1. In a second-level analysis, these individual contrasts were submitted to a one-sample *t*-test across all participants. Based on extensive literature suggesting that left lateral temporal cortex is implicated in housing phonological representations (Booth et al., 2002, 2003, 2004; Prado et al., 2011, 2014), we constrained the brain activation elicited by this contrast within the anatomical left middle and superior temporal gyri (MTG/STG) and considered this combined ROI to represent the storage of verbal representations (Figure 2D).

While different anatomical regions were used to identify the storage of verbal vs. quantity representations, the previous literature on the brain regions involved in *accessing* those representations, especially quantity representations, is less robust. For this reason, we decided to use the same anatomical region, the bilateral frontal cortex (i.e., inferior, middle and superior frontal gyri), to identify the regions involved in accessing verbal and quantity representations. When comparing the brain activation of all dot pairs of the quantity task vs. the control condition within the bilateral frontal cortex, we found three clusters that reached significance: one in the left middle frontal gyrus (MFG; Figure 2C, left), one in right IFG (Figure 2C, middle), and one in right MFG (Figure 2C, right), which were taken as the ROIs involved in accessing quantity representations. This goes in line with Arsalidou’s meta-analyses suggesting that these regions are active for calculation in adults (Arsalidou and Taylor, 2011) and in children (Arsalidou et al., 2018), and for non-symbolic quantity processing (Sokolowski et al., 2017). These regions have also been found to be more active for subtraction as compared to additions (De Smedt et al., 2011; Rosenberg-Lee et al., 2015), for subtractions as compared to a control condition (Kawashima et al., 2004; Evans et al., 2016) and for arithmetic problems reported to be solved by procedures as compared to those reported to be retrieved (Grabner et al., 2009; Polspoel et al., 2017). Given that the role of these three regions in arithmetic processing is not yet clear and that we did not have specific predictions for each area, we treated the three clusters as a single ROI (hereinafter, bilateral MFG/right IFG). To the best of our knowledge, this is the first study that identifies frontal regions involved in quantity processing by means of a localizer task and uses brain activation from these regions to predict subtraction fluency gains.

When constraining the brain activation of all word pairs of the rhyming judgment task vs. the control condition within the bilateral frontal cortex, we found that a cluster in the left inferior frontal gyrus (IFG) was the only one that reached significance (Figure 2E). This finding goes in line with extensive previous evidence suggesting that left IFG is responsible for accessing verbal representations (Poldrack et al., 1999; Rickard et al., 2000; Bookheimer, 2002; Booth et al., 2003, 2004; Prado et al., 2011, 2014; Fedorenko et al., 2012; Andin et al., 2015; Pollack and Ashby, 2017). As shown in Figure 2, the ROIs involved in accessing quantity (2C) and verbal (2E) representations showed no overlap. More information about these combined ROIs is given in Table 2.

**TABLE 2:** Information for regions of interest.

Localizer contrast	Anatomical constraint	K	aal	∼BA	MNI coordinate			*Z*-value	Cluster in Figure 2
					
					*X*	*Y*	*Z*		
									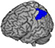
Dot pairs > control	Left IPL/SPL	580	Left IPL/SPL	7/40	−34	−37	38	5.0		
					−40	−41	42	5.0		
					−42	−27	42	4.9	
									
									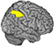
	Right IPL/SPL	286	Right IPL/SPL	7/40	46	−37	54	5.5		
					24	−63	50	5.3		
					30	−53	46	5.1	
									
									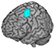
	BilateralIFG/MFG/SFG	202	Left MFG	6	−30	−7	66	5.9		
					−28	−1	58	4.5		
					−26	7	62	3.7	
									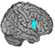
		130	Right IFG oper	44	54	7	26	5.6		
					58	11	18	5.1		
									
									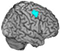
		133	Right MFG	6	32	−1	58	5.5		
									
									
									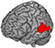
Word pairs > control	Left MTG/STG	495	Left MTG/STG	21/22	−56	−35	2	5.3		
					−42	−61	−2	5.0		
					−50	−67	−2	4.4	
									
									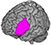
	Bilateral IFG/MFG/SFG	1546	Left IFG	44/45/47	−48	13	26	7.4		
					−38	29	6	6.6		
					−46	27	18	6.3	

*Localizer contrast and anatomical constraint used to create the combined ROIs. Detailed information of the combined ROIs including cluster size (k), corresponding region based on anatomical automatic labeling (aal), approximate Brodmann areas (∼BA), MNI coordinates of the peaks, *Z*-values, and corresponding cluster in Figure 2. MTG/STG, middle and superior temporal gyri; IFG, inferior frontal gyrus; IPL/SPL, inferior and superior parietal lobules; MFG, middle frontal gyrus; SFG, superior frontal gyrus.*

Statistical significance for creating these combined ROIs was defined using Monte Carlo simulations in AFNI’s 3dClustSim program (December, 2015^6^; with SPM’s data smoothness parameters, autocorrelation function [ACF] = 0.45, 4.14, 11.02). 3dClustSim carries out a user-specified number of Monte Carlo simulations of random noise activations at a particular voxel-wise alpha level within a masked brain volume. Following the suggestions made by Eklund et al. (2016) regarding the inflated statistical significance achieved using some packages (i.e., SPM, FSL, and AFNI), we used 3dClustSim’s most recent version (December, 2015). We used 3dFWHMx to calculate the smoothness of the data for every participant, using a spatial ACF, and then averaged those smoothness values across all participants. This averaged smoothness value was then entered into 3dClustSim to calculate the cluster size needed for significance for a given anatomical mask. Cluster sizes of 92, 53, and 53 were needed to reach significance for the bilateral frontal cortex, left MTG/STG and bilateral IPL/SPL anatomical regions, respectively. Clusters exceeding these size thresholds, at a cluster-wise threshold of *p* = 0.05 and voxel-wise threshold of *p* = 0.005, were deemed significant.

#### ROI Analysis

**FIGURE 3:**
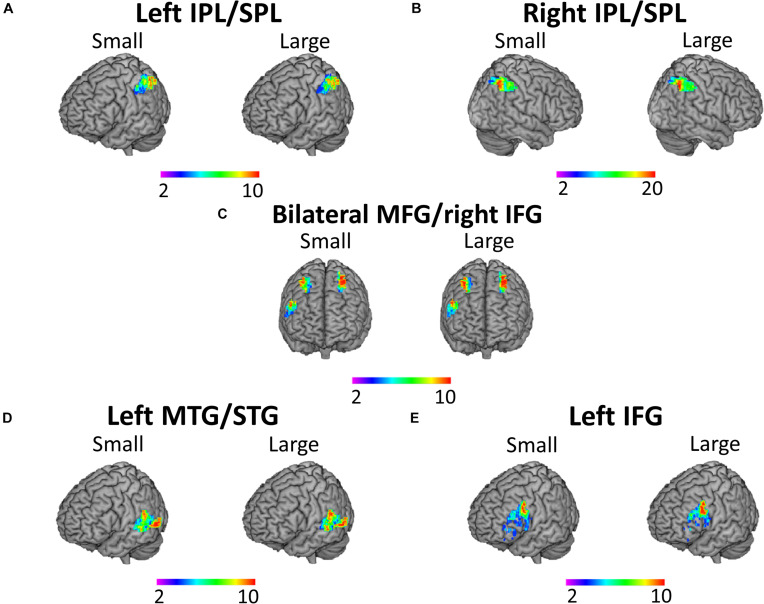
Cluster overlap across participants for activation during subtraction problem-solving. Cluster overlap across participants for the 100 voxels showing maximal activation separately for small and large subtractions in quantity representation ROIs in **(A)** left and **(B)** right IPL/SPL; **(C)** access to quantity representation ROIs in bilateral MFG/right IFG; **(D)** verbal representation ROI in left MTG/STG; and **(E)** access to verbal representation ROI in left IFG. Color bar shows the number of participants showing overlap, from 2 participants shown in purple/blue colors to 10 participants shown in yellow/red colors for all the regions except right IPL/SPL, in which case yellow/red colors indicate 20 participants.

The 100 voxels showing maximal activation for the contrast “small subtractions vs. control” and “large subtractions vs. control” at time 1 were extracted for every participant from each of the five ROIs described above (i.e., left IPL/SPL, right IPL/SPL, bilateral MFG/right IFG, left MTG/STG, and left IFG).^7^ The selection of brain voxels showing maximum activation at the individual level has been suggested to provide higher sensitivity and selectivity, being better able to detect effects and distinguish between conditions (Fedorenko et al., 2010; Nieto-Castañón and Fedorenko, 2012), as compared to traditional group-based analyses that tend to overestimate overlap across participants and underestimate functional specificity (Fedorenko and Kanwisher, 2009). Figure 3 shows the cluster overlap in the five ROIs across participants at time 1, separately for small and large subtractions. Brain activation at time 2 was also extracted from these clusters identified at time 1 in order to study changes in brain activation over time.

Parameter estimates (or β weights) associated with the two contrasts were extracted at the individual level using MarsBars. Subsequently, the extracted data were submitted to SPSS 22 (IBM, SPSS Statistics, IBM Corporation, NY, United States) for statistical testing.

#### Statistical Analyses on Brain Activations During Subtraction Task Solving

Brain activations elicited at time 1 while solving small and large subtractions were separately extracted from the five ROIs (i.e., left IPL/SPL, right IPL/SPL, bilateral MFG/right IFG, left MTG/STG, and left IFG), resulting in 10 variables (i.e., neural problem size effect).

**FIGURE 4:**
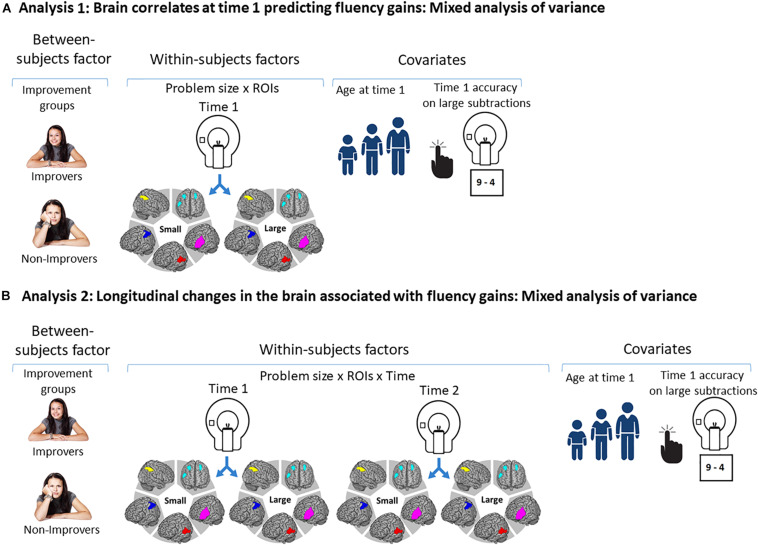
Illustration of the factors included in the statistical analyses. **(A)** Illustration of the between-subjects factors, within-subjects factors, and covariates included in the mixed ANOVAs calculated to study whether improvement groups differed in the brain regions they engaged to solve subtraction problems at time 1. **(B)** Illustration of the between-subjects factors, within-subjects factors, and covariates included in the mixed ANOVAs performed to study the changes in brain activation associated with longitudinal gains in subtraction fluency.

In analysis 1, we studied the role of brain activation at time 1 while solving small and large subtraction problems in predicting math fluency gains. To this aim, we ran a mixed ANOVA including Improvement groups (i.e., improvers; non-improvers) as the between-subjects factor and Problem size (i.e., small, large) × ROI (i.e., L IPL/SPL, R IPL/SPL, bilateral MFG/right IFG, left MTG/STG, and left IFG) at time 1 (i.e., the neural problem size effect) as the within-subjects factors. Participants’ age at time 1 and large subtractions’ accuracy at time 1 were included as covariates. Figure 4A shows an illustration of the between-subjects factor, within-subjects factors, and covariates included in this analysis.

**FIGURE 5:**
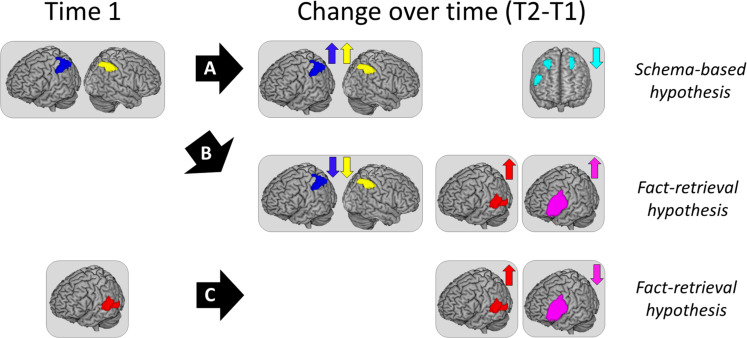
Illustration of the predictions for each hypothesis. **(A)** Arrow indicating the predictions for the Schema-based hypothesis: Initial activation of bilateral IPL/SPL at time 1 followed by increases in brain activation in this region over time, suggesting that children continue to rely on procedures to become more fluent in subtractions. In addition, decreases in bilateral MFG/right IFG would indicate that the implementation of these procedures become more automatic over time, a central claim of the Schema-based hypothesis. **(B)** Arrow indicating a main prediction that would support the Fact-retrieval hypothesis: Initial activation of bilateral IPL/SPL at time 1 is followed by decreases in brain activation in this region and by increases in left MTG/STG activation over time, suggesting that children shift from procedures to retrieval of the solution from long-term memory, a core claim of the Fact-retrieval hypothesis. These changes in brain activation might be accompanied by increases in left IFG over time, considering that the implementation of the retrieval strategy might be effortful in its early stages (Geary et al., 1996a). **(C)** Arrow indicating an alternative prediction that would support the Fact-retrieval hypothesis. Given children 10 years of age use the dominant strategy of retrieval to solve single-digit arithmetic problems (Ashcraft and Fierman, 1982), it may be the case that children have already shifted toward retrieval by the time they were scanned at time 1, which would be consistent with the finding that brain activation in left MTG/STG at time 1 predicts subtraction fluency gains over time. This alternative hypothesis also predicts that brain activation in temporal cortex would increase over time, suggesting that children continue to build their long-term storage of subtraction facts. It is also possible that this change is accompanied by decreases in left IFG activation over time, indicating that retrieval becomes less effortful as the representations in long-term memory become more robust (Prado et al., 2014).

**FIGURE 6:**
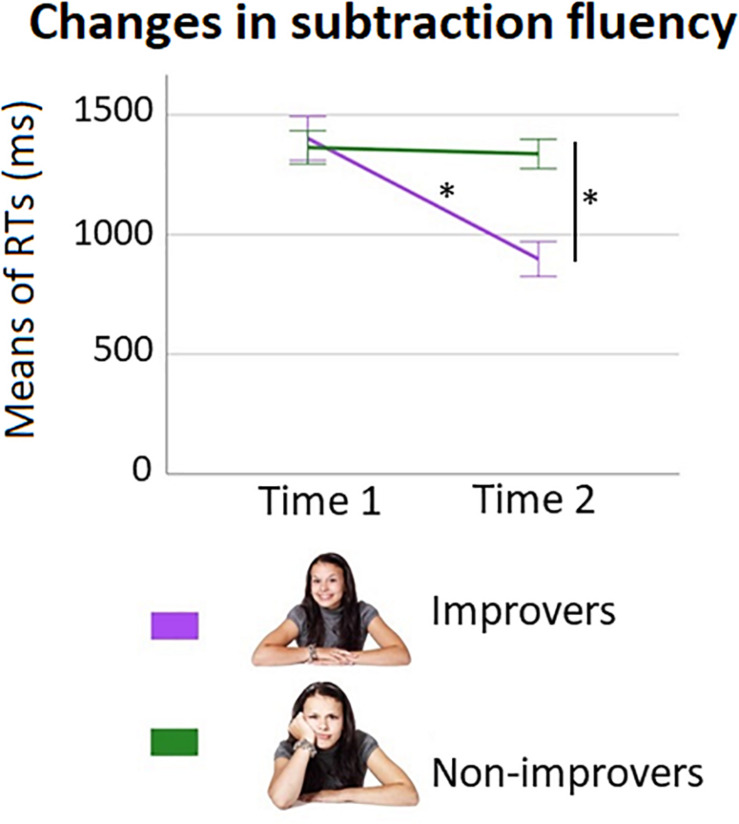
Improvement groups changes in response times (RTs) over time. Changes in RTs (in milliseconds) over time to solve large subtractions separately for improvers and non-improvers. Error bars show standard error of the mean. Asterisks indicate significant differences between groups at time 1 and between time points for improvers.

In analysis 2, we explored the changes over time in brain activation associated with subtraction fluency improvement. We ran a mixed ANOVA including Improvement groups (i.e., improvers; non-improvers) as the between-subjects factor and Problem size (i.e., small, large) × ROI (i.e., L IPL/SPL, R IPL/SPL, bilateral MFG/right IFG, left MTG/STG, and left IFG) × Time (time 1, time 2) as the within-subjects factors. Participants’ age at time 1 and large subtractions’ accuracy at time 1 were included as covariates. Figure 4B shows an illustration of the between-subjects factor, within-subjects factors, and covariates included in this analysis.

Figure 5 shows an illustration of the findings supporting each hypothesis tested in this study, expected at time 1 and expected for the changes in brain activation (time 2 vs. time 1). Finding that brain activation in bilateral IPL/SPL predicts fluency gains would be compatible with both hypotheses given that children may initially rely on parietal-based procedures and continue to do so over time. However, these procedures may become more automatic (i.e., Schema-based hypothesis), or children may initially rely on procedures but later shift toward retrieval (i.e., Fact-retrieval hypothesis). In the first case, illustrated in arrow A in Figure 5, we expected to see increases in bilateral IPL/SPL activation over time, suggesting that children continue to rely on procedures. Critically, we expected to see decreases in bilateral MFG/right IFG over time, suggesting that procedures become more automatic, a central claim of the Schema-based hypothesis. In the second case, illustrated in arrow B in Figure 5, we expected to see decreases in bilateral IPL/SPL and increases in left MTG/STG over time. It is possible that this pattern is accompanied by increases in left IFG activation over time, given that the implementation of retrieval strategies might be effortful in the early stages (Geary et al., 1996a; i.e., Fact-retrieval hypothesis). Finally, there is a third possibility, illustrated in arrow C in Figure 5. Evidence suggests that by 10 years of age retrieval is the dominant strategy to solve single-digit arithmetic problems (Ashcraft and Fierman, 1982), so it is possible that children may have already shifted toward retrieval by the time they were scanned at time 1. In this case, we expected left MTG/STG activation at time 1 to predict subtraction fluency gains and children to show increases in temporal cortex activation over time. These findings would suggest that children continue to build their storage of subtraction facts in long-term memory. This pattern might be accompanied by decreases in left IFG over time, indicating that retrieval becomes less effortful as the representations in long-term memory become more robust (Prado et al., 2014).

#### Whole Brain Analysis

In order to investigate the effects outside our ROIs, we ran a two-sample *t-test* comparing brain activity between improvers and non-improvers at the whole brain (i.e., after excluding ROIs). Following the ROI analysis logic, we focused on (a) brain activation at time 1 by looking at the contrast “large subtractions vs. control at time 1” and (b) changes in brain activation over time by looking at the contrast “large subtractions vs. control time 2 – time 1”. Statistical significance for the whole brain was defined using 3dClustSim. A cluster size of 175 voxels was needed for whole brain significance (ACF values = 0.45, 4.57, 11.14) at a cluster-wise threshold of *p* = 0.05 and a voxel-wise threshold of *p* = 0.005.

## Results

### Localizer Tasks Behavioral Results

We calculated accuracy and RTs (for correctly solved trials) for the rhyming and numerosity judgment localizer tasks, for the participants whose data were used to define ROIs (i.e., *n* = 40; see section “Regions of Interest Definition” for more information). Repeated-measures ANOVAs were performed separately for accuracy and response times, and separately for each localizer task. For the numerosity task, we entered *Difficulty* as the within-subjects factor, which referred to the distance between the number of dots to be compared: easy (12 vs. 36), medium (18 vs.36), and hard (24 vs. 36). As for the rhyming judgment task, we included *Conflict*, which referred to whether orthography was consistent (i.e., non-conflicting) or inconsistent (i.e., conflicting) with the correct answer and *Rhyming*, referring to whether the pair of words rhymed or not, as the within-subject factors. *Post hoc* tests, using Bonferroni correction, were calculated when an effect was found significant.

As for the numerosity judgment task, we found a main effect of *Difficulty* for accuracy [*F*(2,78) = 6.07, *p* = 0.004, *partial* η^2^ = 0.14], showing that accuracy was highest for the easy condition (mean = 90.92, SEM = 1.41), lowest for the hard condition (mean = 86.05, SEM = 1.77), and intermediate for the medium condition (mean = 88.55, SEM = 1.78). The *Difficulty* effect was also significant for the response time analysis [*F*(2,78) = 14.37, *p* < 0.001, *partial* η^2^ = 0.27], and showed fastest response times for the easy condition (mean = 976 ms, SEM = 237), slowest response times for the hard condition (mean = 1061 ms, SEM = 258), and intermediate response times for the medium condition (mean = 1018 ms, SEM = 245).

Regarding the rhyming judgment task, the accuracy analysis showed a main effect of *Rhyming* [*F*(1,39) = 28.58, *p* < 0.001, *partial* η^2^ = 0.42]. Children were more accurate for pairs that rhymed (mean = 90.77, SEM = 1.38) as compared to pairs that did not rhyme (mean = 70.83, SEM = 3.73). The same effect was shown in response times [*F*(1,36)^8^ = 39.95, *p* < 0.001, *partial* η^2^ = 0.53], with children being faster on rhyming pairs (mean = 1185 ms, SEM = 42) than non-rhyming ones (mean = 1364 ms, SEM = 48). We also found a main effect of *Conflict* for accuracy [*F*(1,39) = 64.65, *p* < 0.001, *partial* η^2^ = 0.62], with children being more accurate for non-conflicting (mean = 89.23, SEM = 11.67) than for conflicting pairs (mean = 72.37, SEM = 17.50). The same main effect of *Conflict* was found for response times [*F*(1,36) = 18.86, *p* < 0.001, *partial* η^2^ = 0.34], with children taking longer to respond to conflicting (mean = 1315 ms, SEM = 293) than to non-conflicting pairs (mean = 1223 ms, SEM = 246). The *Rhyming* × *Conflict* interaction was also significant for accuracy [*F*(1,39) = 24.17, *p* < 0.001, *partial* η^2^ = 0.38]. While the comparisons across all conditions were significant (all *p-*Values below.005), the interaction showed that the non-rhyming condition with conflicting orthography (O + P−) was the hardest. The *Rhyming* × *Conflict* interaction was also significant for response times [*F*(1,36) = 8.23, *p* = 0.007, *partial* η^2^ = 0.19]. The interaction was due to a significant difference between the conflicting and non-conflicting conditions among the non-rhyming pairs (*p* < 0.001; O + P− and O-P-), but a non-significant difference between conflicting and non-conflicting conditions among the rhyming pairs (*p* = 0.22; O + P+, and O−P +).

### Subtraction Task Behavioral Results

#### Whole Sample Performance

We calculated accuracy and means of RTs (for correctly solved trials) separately for small and large subtractions, for every participant.

We calculated a repeated measures ANOVA for accuracy including Time (i.e., time 1, time 2) and Problem size (i.e., small, large) as within-subjects factors. *Post hoc* tests, using Bonferroni correction, were calculated when an effect was found significant. We found a main effect of Time [*F*(1,45) = 23.15, *p* < 0.001, *partial*η^2^ = 0.34] and a main effect of Problem size [*F*(1,45) = 25.67, *p* < 0.001, *partial*η^2^ = 0.36], but no Time × Problem size interaction [*F*(1,45) = 0.49, *p* = 0.49, *partial*η^2^ = 0.01]. The Time effect showed that, across problem sizes, children were more accurate at time 2 (mean = 89.53, SEM = 1.24) as compared to time 1 (mean = 80.50, SEM = 2.08; *p* < 0.001). The Problem size effect showed that, across time points, children were more accurate solving small (mean = 87.70, SEM = 1.28; *p* < 0.001) as compared to large (mean = 82.33, SEM = 1.74) subtractions.

We then calculated a repeated measures ANOVA for means of RTs including Time (i.e., time 1, time 2) and Problem size (i.e., small, large) as within-subjects factors. We found a main effect of Time [*F*(1,45) = 49.33, *p* < 0.001, *partial*η^2^ = 0.52], and a main effect of Problem size [*F*(1,45) = 44.46, *p* < 0.001, *partial*η^2^ = 0.50], but no Time × Problem size interaction [*F*(1,45) = 0.005, *p* = 0.94, *partial*η^2^ = 0.00]. The Time effect showed that, across problem sizes, children were faster at time 2 (mean = 1049 ms, SEM = 52) as compared to time 1 (mean = 1313 ms, SEM = 54; *p* < 0.001). The Problem size effect showed that, across time points, children were faster to solve small (mean = 1113 ms, SEM = 48; *p* < 0.001) as compared to large (mean = 1250 ms, SEM = 52) subtractions.

#### Improvement Groups’ Performance

We then explored children’s performance in large subtractions depending on improvement groups (see section “Improvement Groups” for a description of how groups were formed). This confirmatory analysis was carried out to test whether groups showed the expected pattern of behavioral changes over time.

We calculated a repeated-measures ANOVA for accuracy entering Time (Time 1; Time 2) as the within-subjects factor and Improvement groups (improvers, non-improvers) as the between-subjects factor. The same ANOVA was calculated for RTs.

As for accuracy, we found a significant main effect of Time [*F*(1,44) = 16.72, *p* < 0.001, *partial*η^2^ = 0.28], but no Time × Improvement group interaction [*F*(1,44) = 0.62, *p* = 0.44, *partial*η^2^ = 0.01]. The main effect of Time showed that, regardless of improvement group, all children became more accurate [*t*(45) = 5.66, *p* < 0.001]. More detailed information about groups’ performance is given in Table 3. The main effect of Time did not reach significance when age at time 1 was entered as a covariate in the ANOVA [*F*(1,43) = 1.71, *p* = 0.20, *partial*η^2^ = 0.04].

**TABLE 3:** Performance on large subtractions solved inside the scanner.

	Whole (*n* = 46)	Improvers large (*n* = 23)	Non-improvers large (*n* = 23)
Accuracy T1	77.5 (17.1)	79.1 (15.7)	75.9 (18.6)
Accuracy T2	87.2 (10.7)	86.9 (11.5)	87.4 (10.0)
Accuracy change	9.7 (16.0)	7.8 (13.7)	11.5 (18.1)
RTs T1	1382 (387)	1401 (441)	1363 (333)
RTs T2	1117 (389)	898 (350)	1336 (294)
RTs change	−265(317)	−503(216)	−27(202)

*Means of response times (RTs; in milliseconds), and accuracy (standard deviation in parentheses) at time 1 (T1) and time 2 (T2) and change (time 2 − time 1) for the whole sample (*n* = 46), for improvers (*n* = 23), and for non-improvers (*n* = 23).*

Regarding RTs, the main effect of Time [*F*(1,44) = 73.97, *p* < 0.001, *partial*η^2^ = 0.63] was significant. As expected, based on the definition of the improvement groups, the Time × Improvement group interaction was also significant [*F*(1,44) = 59.74, *p* < 0.001, *partial*η^2^ = 0.58]. The interaction showed that the improvers had a significant decrease in RTs over time [*t*(22) = 11.17, *p* < 0.001], whereas the non-improvers did not [*t*(22) = 0.64, *p* = 0.53]. Groups differed in RTs at time 2 [*t*(44) = −4.60, *p* < 0.001], but not at time 1 [*t*(44) = 0.33, *p* = 0.75]. More detailed information about groups’ performance is given in Table 3. Figure 6 shows the changes over time in RTs for improvers and non-improvers. Results were consistent if age at time 1 was entered as a covariate in the ANOVA (i.e., Time × Improvement group interaction: *F*(1,43) = 61.06, *p* < 0.001, *partial*η^2^ = 0.59).

### fMRI Results

#### Improvers Showed a Larger Neural Problem Size Effect in Bilateral Parietal Cortex at Time 1

**FIGURE 7:**
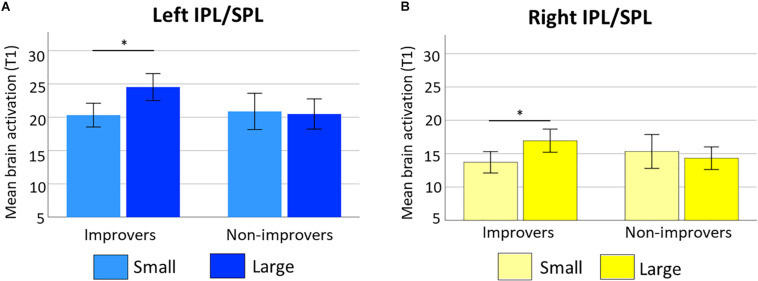
Neural problem size effect in bilateral parietal cortex at time 1 for improvement groups. Bar chart show brain activation in **(A)** left IPL/SPL and **(B)** right IPL/SPL for small (i.e., lighter colors) and large (i.e., darker colors) subtractions, for improvers and non-improvers. Error bars show standard error of the mean. Asterisks indicate significant differences between problem sizes in left and right IPS for improvers.

The analysis of brain activation at time 1 showed a significant ROI × Problem size × Improvement groups interaction [*F*(3,138) = 2.66, *p* = 0.04, *partial*η^2^ = 0.06, Greenhouse-Geisser ε = 0.82]^9^. We explored the three-way interaction with pairwise comparisons using Bonferroni correction to control for multiple comparisons. This analysis showed differences in the left IPL/SPL [*t*(22) = −4.41, *p* = 0.001] and right IPL/SPL [*t*(22) = −3.57, *p* = 0.01] between small and large subtractions only for improvers. Figure 7 shows the differences in brain activation in the left (A) and right (B) IPL/SPL between small and large subtractions for the improvers and non-improvers groups. The two group did not differ in bilateral MFG/R IFG (*p* = 0.47), left MTG (*p* = 0.26), or left IFG (*p* = 0.17) at time 1. The non-improvers showed no neural problem size effect in any of the ROIs (all *p-*Values above 0.25).

#### Improvers Decreased Activation for Large Subtractions in Both Parietal and Frontal ROIs Over Time

**FIGURE 8:**
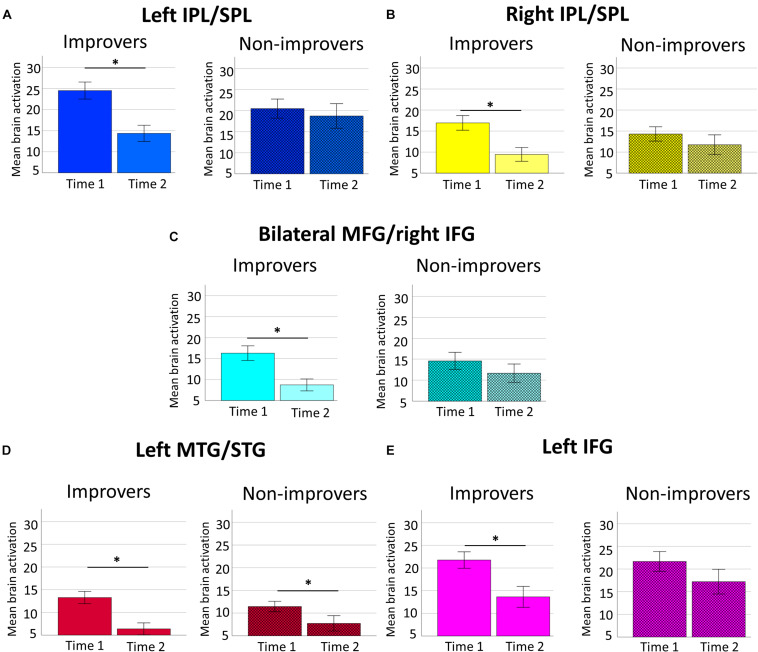
Changes in brain activation over time for large subtractions for improvers and non-improvers. Changes in brain activation in **(A)** left IPL/SPL, **(B)** right IPL/SPL, **(C)** bilateral MFG/right IFG, **(D)** left MTG/STG, and **(E)** left IFG for children showing improvement (i.e., plain bars) and non-improvement (i.e., patterned bars). Error bars show standard error of the mean. Asterisks indicate significant differences between time points in all ROIs for improvers and in left MTG/STG for non-improvers.

The ANOVA showed a Time × ROI × Problem size × Improvement groups interaction [*F*(3,126) = 2.76, *p* = 0.04, *partial*η^2^ = 0.06, Greenhouse-Geisser ε = 0.75].^10^ Pairwise comparisons using Bonferroni correction showed a different pattern of changes in brain activation over time depending on problem size. For small subtractions, both groups showed significant decreases in brain activation over time in all ROIs (all *p-*Values below.02). As for large subtraction problems, improvers showed a significant decrease over time in all ROIs (all *p-*Values equal or below.001), whereas non-improvers showed significant decreases over time only for left MTG/STG (*p* = 0.005), but not for left IPL/SPL (*p* = 0.40), right IPL/SPL (*p* = 0.13), bilateral MFG/right IFG (*p* = 0.07) or left IFG (*p* = 0.054). Figure 8 illustrates changes over time in brain activation for large subtraction problems for improvers (i.e., plain bars) and non-improvers (i.e., patterned bars).

#### Evidence for the Efficiency of Numerical Procedures: A Exploratory Analysis of the Improvers

**FIGURE 9:**
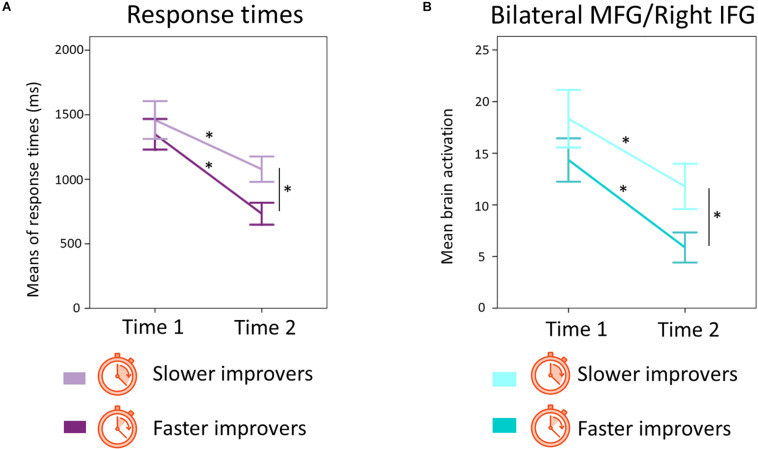
Changes in RTs and changes in bilateral MFG/right IFG activation for the subgroup of improvers that ended up being faster (i.e., *faster improvers*) or slower (i.e., *slower improvers*) at time 2. **(A)** Changes over time in response times for the subgroup of faster and slower improvers. Error bars show standard error of the mean. Asterisks confirm significant differences between time points for both subgroups and significant differences in response times between subgroups at time 2. **(B)** Changes over time in bilateral MFG/right IFG activation for the subgroups of faster and slower improvers. Error bars show standard error of the mean. Asterisks indicate significant differences between time points for both subgroups and significant differences in brain activation between subgroups at time 2.

We aimed to further explore the idea of efficiency of numerical procedures by more closely looking at the improvers group. Considering our finding, showing decreased bilateral MFG/right IFG activation over time for improvers, it would be reasonable to expect greater decreases in these regions for children becoming faster over time, even among the improvers (*n* = 23), providing further evidence for the automaticity in the implementation of procedures. To this aim, participants in the improvers group were split into two subgroups: *slower* improvers (*n* = 11) and *faster* improvers (*n* = 12), based on the same procedure used to define the improvers vs. non-improvers and described in section “Improvement Groups”. As shown in Figure 9A, the *slower* improvers group [*t*(10) = 7.50, *p* < 0.001] and the *faster* improvers group [*t*(11) = 10.77, *p* < 0.001] significantly decreased in response times over time, but they differed in how fast they solve problems at time 2 [*t*(21) = 2.66, *p* = 0.01].

We also ran a student *t-test* comparing bilateral MFG/right IFG brain activation between *slower* and *faster* improvers at each time point. As shown in Figure 9B, results showed that groups differed in brain activation at time 2 [*t*(21) = −2.27, *p* = 0.03], but not at time 1 [*t*(21) = −1.16, *p* = 0.26], with the *faster* subgroup showing less bilateral MFG/right IFG activation than their *slower* counterparts at time 2. Both the *slower* [*t*(10) = 3.98, *p* = 0.003] and the *faster* [*t*(11) = 5.32, *p* < 0.001] improvers subgroups significantly decreased brain activation in this area over time.

#### Whole Brain Results

Three clusters (shown in Supplementary Figure 1) reached significance for the contrast “large subtractions vs. control time 2 – time 1”, showing greater activation for non-improvers as compared to improvers. More specific information about these clusters is provided in Table 4. No cluster reached significance for the contrast “large subtractions vs. control time 1”.

**TABLE 4:** Whole brain results.

K	MNI coordinate			Z-score	∼BA	Anatomical region
	
	X	Y	Z			
176	−2	−81	22	3.93	17	left cuneus and left calcarine
	−2	−91	18	3.48		
	2	−83	6	2.78		
194	32	−33	38	3.70	2, 3, 40	right postcentral and right supramarginal gyrus
	40	−35	46	3.49		
	44	−23	42	3.08		
215	−38	−53	6	3.93	37	left middle occipital cortex
	−36	−65	2	3.63		
	−36	−67	22	3.50		

*Clusters showing significant activation at the whole brain level for large subtraction problems as compared to the control condition at time 2 as compared to time 1 for the non-improvers as compared to the improvers.*

## Discussion

Despite the crucial role that mathematics plays in our society for personal and professional development, and the importance that developing math fluency has in the acquisition of more advanced mathematics (Geary, 1994; Price et al., 2013), the neurocognitive mechanisms associated with improvement in behavioral fluency are poorly understood. While there is consensus in the literature that children show a shift toward retrieval for operations such as multiplication (Ashcraft, 1982; Dehaene and Cohen, 1995), it is not yet clear how fluency is achieved in subtraction. Two hypotheses have been formulated to explain subtraction fluency development. According to the Fact-retrieval hypothesis, children become fluent in single-digit subtractions by shifting from procedures to the retrieval of the solutions from declarative long-term memory (Ashcraft, 1982; Siegler, 1987). The Schema-based hypothesis, on the other hand, claims that children achieve subtraction fluency by means of procedures that become automatic over development (Baroody, 1983; Fayol and Thevenot, 2012). Given that both hypotheses make the same predictions regarding changes in RTs but differ in the mechanisms considered to be responsible for that change, and given that automatic processes seem to be easily confounded with and reported as retrieval (Fayol and Thevenot, 2012), neither RTs nor self-reported measures have been able to adjudicate between these two hypotheses. Within this context, fMRI can help by investigating (a) whether engagement of verbal or quantity brain areas early on (i.e., time 1) predict longitudinal gains in subtraction fluency; (b) whether longitudinal fluency gains are associated with changes in verbal or quantity brain activation. Importantly, the aim of this study was to assess differences in the neurocognitive mechanisms recruited by children differing in fluency but showing similar levels of accuracy on the experimental task.

### Modulation of Parietal Cortex by Problem Size at Time 1 Predicts Longitudinal Subtraction Fluency Improvement

When examining the role of brain activation at time 1 in predicting longitudinal gains in subtraction fluency we found that improvers showed a larger neural problem size effect in bilateral IPS at time 1, with greater activation for large subtractions as compared to small ones. These results are consistent with Prado et al. (2014)’s cross-sectional evidence showing grade-related increases in parietal cortex for solving subtractions with more years of math instruction. Prado et al. (2014)’s and our results both support the involvement of quantity but not verbal regions for subtraction learning. However, there were some differences between studies. First, the covariate of interest in our study was how much children improved from time 1 (i.e., sample in Prado’s papers) to time 2, with age being controlled for. In contrast, Prado et al. (2014) study included grade (i.e., second through eighth), which is highly correlated with age, as the predictor of interest. Second, while Prado et al. (2014) found the effects only for small subtractions, we found them for large subtractions. Third, while Prado et al. (2014) found the effects in the right posterior superior parietal lobule (PSPL), we found them in bilateral IPS. While the IPS plays a role in representing quantities (Dehaene et al., 2003; Piazza et al., 2004), the PSPL has been associated with visuo-spatial attentional processes in children (Krinzinger, 2011) and adults (Simon et al., 2002). Previous results have suggested overlapping patterns of activity in PSPL for addition and subtractions and shifts of visuo-spatial attention (Knops et al., 2009), like the ones needed to estimate the position along a mental number line (Berteletti et al., 2014). Prado et al. (2014) concluded that these visuo-spatial shifts seemed to be sufficient for solving small subtractions, while solving large ones would require more involvement of quantity mechanisms in IPS. Our results confirm Prado’s predictions by showing that engaging these quantity mechanisms in IPS, early on, explained longitudinal gains in subtraction fluency.

### Interpretation of the Decreases in Parietal Activation Over Time as Supporting the Schema-Based Hypothesis

The fact that parietal cortex at time 1 was the only ROI that predicted subtraction fluency improvement does not distinguish between the Schema-based and Retrieval-based hypotheses. It could be the case that this early parietal engagement, suggesting procedural use, is replaced by the retrieval of the solution from memory, in which case we should see a shift in brain activation from parietal to temporal regions. We hypothesized that this could be accompanied by increases in left IFG, given that the implementation of retrieval strategy might be effortful in young children (Geary et al., 1996a). Alternatively, it might be the case that the use of procedures is not replaced, but becomes more efficient over time, in which case we would see increases in parietal cortex over time, suggesting that children continue to rely on procedures to develop their fluency. This should be accompanied by decreases in bilateral MFG/right IFG over time, suggesting that procedures become more automatic, a core claim of the Schema-based hypothesis.

The analysis of longitudinal changes in brain activation showed that children who improved in subtraction fluency decreased activation in bilateral IPL/SPL over time. Previous evidence has suggested that *less* activation for a given level of proficiency represents more efficient use of certain brain regions (Prat et al., 2007; Prat and Just, 2011). Several fMRI studies have found that more skilled or highly trained individuals show less brain activation as compared to controls (Rypma and D’Esposito, 1999; Krings et al., 2000; Welcome and Joanisse, 2012). In addition, decreased activation in different brain regions has been found after practice with visuomotor association tasks (Büchel et al., 1999), visuospatial WM (Garavan et al., 2000), verbal WM (Hempel et al., 2004), Tower of London (Beauchamp et al., 2003), or counting Stroop tasks (Bush et al., 1998) in which participants became faster with training. In the field of math cognition, previous work has shown decreased brain activation for perfect performers (i.e., 100% accuracy) as compared to imperfect performance (i.e., 78%-96% accuracy; Menon et al., 2000), suggesting that after a certain level of expertise is achieved, the brain can achieve the same results with fewer resources. Within this context, and considering that the reductions in parietal cortex activation were unique to the improvers group, we interpret our findings as showing that gains in subtraction fluency is associated with a more efficient recruitment of parietal cortex by calculation procedures. The fact that non-improvers continue to engage parietal cortex over time is consistent with previous evidence showing greater bilateral parietal activation for children with developmental dyscalculia solving a subtraction task as compared to an addition task (Rosenberg-Lee et al., 2015). This finding is also consistent with evidence showing that 8 weeks of one-to-one math tutoring resulted in significant reductions in overactivation of bilateral IPS (among other regions) in children with math learning disabilities (Iuculano et al., 2015).

### Decreases in Bilateral MFG/Right IFG Supports the Schema-Based Hypothesis

Our finding of a reduction in brain activation in bilateral MFG/right IFG supports the Schema-based hypothesis and suggests that processes occurring in these areas become more automatic over time. Decreases in bilateral parietal and frontal regions were interpreted as evidence for procedures becoming more efficient in a training study with adults that found untrained subtractions engaged bilateral IPS and bilateral IFG as compared to trained ones (Ischebeck et al., 2006). Less activation in MFG over development has also been observed in a cross-sectional study in 8−19-year-old children solving additions and subtractions (Rivera et al., 2005), in a longitudinal study of 6th to 7th-grade children solving two-digit subtractions (Artemenko et al., 2018), and in adults solving arithmetic problems as compared to children (Kawashima et al., 2004; Kucian et al., 2008). Our finding adds to this evidence by showing that frontal regions involved in quantity processing, as identified with a numerosity judgment localizer task, decreased activation with improvement in subtraction fluency and support the hypothesis of increased automaticity in accessing procedures.

### Underlying Mechanisms Explaining the Automaticity of Procedures

Our findings support previous studies suggesting that, at least for subtractions, developing fluency involves procedures becoming more automatic over time (Baroody, 1983; LeFevre et al., 2006; Fayol and Thevenot, 2012). Using a priming paradigm, Fayol and Thevenot (2012) tested whether solving additions, subtractions, and multiplications mobilized a procedural component or were solved by retrieval. They tested whether procedures were pre-activated as soon as individuals see a sign (i.e., +, -, x), presented before the arithmetic problem indicating the upcoming operation. They found that solving additions and subtractions was facilitated when the operation sign was presented 150 ms before the operands and that this effect was operation-specific. They inferred that abstract procedures were primed by the presentation of the sign, subsequently helping with solving the problems. The presentation of the multiplication sign had no facilitation effect on solving the problems, confirming the hypothesis that they did not rely on procedures. Moreover, subtractions were not solved slower than multiplications, suggesting that procedures could be as fast as retrieval. In a similar study, Mathieu’s et al. (2016) presented the first operand and the operator in the center of the screen, while the second operand was presented either in the left or the right of the screen. They found that additions were solved faster when the second operand appeared to the right of the screen whereas subtractions were solved faster when the operand was presented to the left. No effect was found for multiplication. They interpreted these findings as suggesting that solving additions and subtractions activated procedures consisting of rightward and leftward shifts of attention, respectively, along a mental number line. Furthermore, in a study of the neural correlates of these effects, Mathieu’s et al. (2018) found greater activation in brain regions supporting the orientation of spatial attention, including right posterior superior parietal lobule (PSPL), when participants were presented with the “+” sign as compared to the “x” one. They interpreted that the operation-priming effect shown by Fayol and Thevenot (2012) was due to arithmetic symbols evoking spatial mechanisms that would, in turn, lead to facilitation of performance for that operation.

While previous studies from our lab have interpreted grade-related findings in the PSPL for small subtractions as suggesting visuo-spatial attentional shifts, this interpretation seems less likely to explain our findings in the IPS, a brain region well known for its role in quantity representation (Dehaene et al., 2003; Piazza et al., 2004). We believe that reliance on quantity-based procedures becomes more automatic because these representations in parietal cortex are refined over development (Suárez-Pellicioni and Booth, 2018). Several studies have shown that with experience to symbolic mathematics, children develop more precise representation of quantities (Ansari, 2008; Mussolin et al., 2014; Matejko and Ansari, 2016). If quantity representations are more refined, children are better able to implement calculation procedures more efficiently, requiring less parietal activation. More precise quantity representations would also explain the decreases in bilateral MFG/right IFG regions over time, suggesting calculation procedures become less effortful.

### The Case of Addition: An Ongoing Debate

A consensus has not yet been reached regarding whether the Fact-retrieval or the Schema-based hypotheses better explain arithmetic fluency development for arithmetic problems involving addition. As mentioned above, Fayol and Thevenot (2012), Mathieu’s et al. (2018, 2016) results suggest that solving both addition and subtraction problems rely on procedures. Barrouillet and Thevenot (2013), showed that response times monotonically and linearly increased when addends were incremented by one, a finding they argued is not consistent with retrieval use, but rather points to adults relying on fast procedures to solve additions. Uittenhove et al. (2016) also argued that it was difficult to interpret the high variability in response times to addition problems resulting from a one-step direct retrieval process. Finally, Thevenot et al. (2016) aimed to challenge previous evidence suggesting that by 10 years old children already rely on retrieval to solve single-digit arithmetic problems (Ashcraft and Fierman, 1982). Their analysis of 10-year-old children’s response time patterns to a single-digit addition production task was compatible with shifting from slow to fast counting procedures but not with a shift toward retrieval.

A very recent study used EEG to try to clarify between the fact-retrieval and the schema-based hypotheses by administering adults a single-digit addition and multiplication production task. Their analyses of theta, lower alpha, and upper alpha frequencies showed higher evidential strength for similar EEG activity between very small additions (i.e., operands between 1 and 4) and multiplication problems, suggesting that very small additions are solved through fact retrieval, and supporting the Fact-retrieval hypothesis (Grabner et al., 2020). Other studies investigating subtraction problem solving using fMRI have reported evidence suggesting that additions are solved through retrieval, with the hippocampus playing a potentially important role in memory formation for these facts (Cho et al., 2012). Using a multivariate analysis, Cho et al. (2011) found differences in neuronal activity patterns between 7- to 9-year-old children that were classified as retrievers vs. counters when solving a single-digit addition task, with the highest classification rates being observed in the bilateral hippocampus. Greater hippocampal activation in children was found for additions as compared to subtractions by De Smedt et al. (2011). Qin et al. (2014) longitudinal study showed that 7-to 9-years-old children showed increases in hippocampus activation and decreases in prefrontal-parietal activation during addition problem solving, suggesting a transition from counting to retrieval (Qin et al., 2014). Rosenberg-Lee et al. (2018) found increases in hippocampus and decreases in fronto-parietal activity when children solved a single-digit addition verification task after they completed an 8-week number and arithmetic training. In summary, studies suggest an important role of the hippocampus for addition, so future studies need to address the role of this brain area in distinguishing between the Schema-based and Fact-retrieval hypotheses.

### No Evidence Supporting the Fact-Retrieval Hypothesis in Our Study

The Fact-retrieval hypothesis would have been supported by the finding of greater activation in left MTG/STG at time 1 predicting gains in subtraction fluency or brain activation shifting from parietal to temporal cortex over time. We found no such effects. Our results showed that all children, *regardless of improvement*, showed decreased brain activation in verbal regions over time for large subtractions. The lack of a problem size effect in verbal regions at the first time point and the fact that brain activation in this region decreased over time regardless of improvement argues against the Fact-retrieval hypothesis, suggesting that a shift toward retrieval is not the underlying mechanism for gains in subtraction fluency. Even when looking at the whole brain, no significant differences between improvement groups were found in any region at time 1. For the changes in brain activation over time, greater activation was found for non-improvers as compared to improvers in a region sometimes reported in studies looking at retrieval, the supramarginal gyrus (e.g., Lee, 2000; Rivera et al., 2005). While the exact role of supramarginal gyrus in arithmetic processing is not yet clear, the finding suggests that engaging this region is actually associated with a lack of improvement in fluency. We found no significant brain activation in regions considered to play a role in memory formation, such as the hippocampus (e.g., Cho et al., 2011) or in other regions reported to be activated (or deactivated) when retrieving, such as the angular gyrus (e.g., De Smedt et al., 2011). In line with Thevenot et al. (2016), our study argues against previous evidence suggesting that by the time children are 10-years-old, they rely on retrieval to solve single-digit arithmetic problems (Ashcraft and Fierman, 1982), suggesting instead that different operations recruit distinct neural networks (Arsalidou and Taylor, 2011; Rosenberg-Lee et al., 2011), even for single-digit problems.

### Educational Relevance and Conclusion

Our fMRI study has filled a gap in the literature by providing evidence that early reliance on brain areas implicated in quantity representation is an important predictor explaining gains in subtraction fluency in children. This finding supports the Schema-based hypothesis, and is consistent with previous behavioral (Baroody, 1983; LeFevre et al., 2006; Fayol and Thevenot, 2012) and fMRI (Prado et al., 2011, 2014; Evans et al., 2016) evidence suggesting that children do not rely on retrieval to solve subtractions, but that procedures become more automatic with skill development to support this operation (Fayol and Thevenot, 2012; Barrouillet and Thevenot, 2013). Our study constitutes an example of the utility of neuroimaging to provide important information in order to answer educationally relevant questions.

In our study, we did not give children any instruction in the kind of strategy they should use to solve the task, so it is likely that individuals used different strategies. However, it was the children who relied on quantity mechanisms by engaging parietal cortex early in development the ones who showed greater fluency 2 years later. This finding suggests that the engagement of parietal-based calculation strategies should be encouraged in the classroom to solve subtraction problems. We argue that calculation practice over the course of formal math education will lead to subtractions becoming more automatic. We found no evidence suggesting that the rote memorization of subtraction facts should be encouraged in school.

According to Siegler’s adaptive strategy choice model (Siegler and Shipley, 1995), arithmetic strategies are chosen depending on their efficiency. One reason why relying on procedures to solve subtractions might be more efficient than retrieval has to do with their non-commutative nature. While additions and multiplications are commutative so, for example, 3 + 6 and 6 + 3 could share common memory nodes (Rickard and Bourne, 1996), subtraction is not. Using retrieval might not be efficient for solving subtractions because children would have to store in memory twice the number of subtraction facts (i.e., 6−3 = 3, but 3−6 = −3). As suggested by Campbell and Xue (2001), there should be greater retrieval interference for subtraction facts, making retrieval less efficient for this operation and promoting the use of procedures.

We cannot rule out the possibility that the effects we found in the brain are the consequence of the way subtractions are taught in the United States. Math curriculum in North America emphasizes conceptual understanding over fact mastery (Geary et al., 1996b), with subtractions usually being taught by using counting strategies or inverse addition (Geary et al., 1993; LeFevre et al., 2006), and engaging brain regions involved in finger representations (Berteletti and Booth, 2015a). According to Siegler’s distribution of associations model (Siegler and Jenkins, 1989), with experience, certain problems become associated with certain strategies, as do problems with answers. If a problem is consistently associated with a given strategy, then the association between them can be even stronger than the problem-solution association, leading to the application of the most frequently used strategy. Children may also rely more consistently on procedures for subtractions to avoid the switching cost associated with mixing strategies (Lemaire and Lecacheur, 2010). Considering imaging evidence showing that the method of learning arithmetic has a direct impact on the brain (Delazer et al., 2005), it is possible that these teaching differences across countries could play an important role in supporting the Fact-retrieval vs. Schema-based hypotheses. Future studies comparing students from countries having a different emphasis on retrieval should be carried out to test this hypothesis.

## Data Availability Statement

The datasets generated for this study are available on request to the corresponding author.

## Ethics Statement

The studies involving human participants were reviewed and approved by Northwestern University Institutional Review Board. Written informed consent to participate in this study was provided by the participants’ legal guardian/next of kin.

## Author Contributions

JB conceptualized and designed the project and supervised the data collection. MS-P and JB formulated the research question. IB contributed to data collection. MS-P analyzed the data and wrote the first draft of the manuscript. All authors contributed to the interpretation of the results, revised the manuscript, and approved the final version for publication.

## Conflict of Interest

The authors declare that the research was conducted in the absence of any commercial or financial relationships that could be construed as a potential conflict of interest.

## Footnotes

1 The following participants were included in this study: 2, 5, 6, 7, 8, 9, 10, 11, 12, 13, 16, 20, 22, 27, 34, 35, 36, 40, 44, 45, 48, 49, 50, 53, 54, 56, 57, 59, 60, 65, 67, 69, 70, 71, 75, 76, 77, 83, 86, 89, 90, 93, 95, 96, 103, and 106.

2 http://surfer.nmr.mgh.harvard.edu/optseq/

3 www.fil.ion.ucl.ac.uk/spm

4 https://cibsr.stanford.edu/tools/human-brain-project/artrepair-software.html

5 Participants 50, 56, 65, 71, 83, and 96 were excluded from the ROI definition.

6 http://afni.nimh.nih.gov/

7 Note that the variable of interest in this study is the extent to which participants relied on quantity representation mechanisms, indicating the use of calculation-based strategies, so we extracted brain activation *during the subtraction task* (i.e., top 100 voxels) from ROIs in parietal cortex that were identified during the numerosity judgment task using the contrast “all dots vs. control”. In contrast, in Suárez-Pellicioni and Booth (2018), we explored the role of quantity representation at time 1 in predicting math fluency gains, with parietal activation *during the Numerosity judgment task* (i.e., contrast “hard vs. easy”) being our covariate of interest.

8 The difference in degrees of freedom is due to 3 participants having no correct responses for one of the four conditions, so response times could not be calculated, resulting in missing data.

9 Results remained significant if the top 50 [*F*(3,136) = 3.00, *p* = 0.03, *partial*η^2^ = 0.07, Greenhouse-Geisser ε = 0.81] or top 200 [*F*(3,137) = 3.60, *p* = 0.01, *partial*η^2^ = 0.08, Greenhouse-Geisser ε = 0.82] voxels were selected instead of the top 100.

10 Results remained significant if the top 50 [*F*(3,125) = 3.10, *p* = 0.03, *partial*η^2^ = 0.07, Greenhouse-Geisser ε = 0.75] or the top 200 [*F*(3,125) = 2.75, *p* = 0.046, *partial*η^2^ = 0.06, Greenhouse-Geisser ε = 0.75] voxels were selected instead of the top 100.
