# Supplementary material for: Early Engagement of Parietal Cortex for Subtraction Solving Predicts Longitudinal Gains in Behavioral Fluency in Children
Source: Front Hum Neurosci. 2020 May 26;14:163. doi: 10.3389/fnhum.2020.00163 (PMC7264824; doi:10.3389/fnhum.2020.00163)

Supplementary Figure 1. Whole brain results. Clusters showing significant activation at the whole brain level for large subtraction problems as compared to the control condition at time 2 as compared to time 1 for the non-improvers as compared to the improvers.

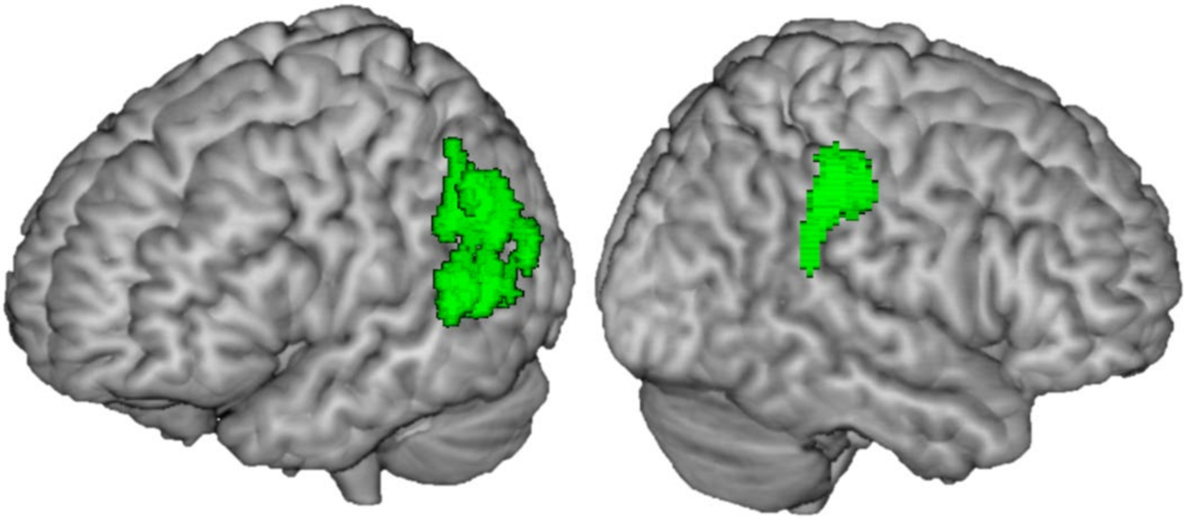

Supplement: Supplementary file 1 [file Image_1.pdf]
